# Comparaison de deux scores pronostiques dans les hémorragies digestives hautes non variqueuses dans un centre hospitalier d'Antananarivo

**DOI:** 10.11604/pamj.2013.16.126.2089

**Published:** 2013-11-29

**Authors:** Aurélia Rakotondrainibe, Thierry Pierre Randrianambinina, Harifetra Mamy Richard Randriamizao, Fanjandrainy Rasoaherinomenjanahary, Mialimanana Joël Randriamiarana, Luc Hervé Samison

**Affiliations:** 1 Service de Réanimation Chirurgicale. Centre Hospitalo-Universitaire d'Antananarivo, Joseph Ravoahangy Andrianavalona. Madagascar; 2 Service de Chirurgie viscérale B. Centre Hospitalo-Universitaire d'Antananarivo, Joseph Ravoahangy Andrianavalona. Madagascar

**Keywords:** Antananarivo, Hémorragie digestive haute, Non variqueuse, Score de Glasgow Blatchford, Score de Rockall initial, Antananarivo, Upper gastrointestinal haemorrhage, non-variceal, Glasgow Blatchford score, Initial Rockall score

## Abstract

Il est actuellement recommandé de stratifier les hémorragies digestives hautes, en fonction de leur risque, par des scores pronostiques. L'objectif de cette étude a été d'appliquer ces scores en vue de les comparer, dans un centre hospitalier malgache. Etude prospective sur 419 patients, sur une période entre janvier et décembre 2011, au Centre Hospitalo-Universitaire d'Antananarivo. Les scores de Rockall initial et de Blatchford ont été calculés pour chaque patient présentant une hémorragie digestive haute non variqueuse dont les dossiers été complets, avec notamment la valeur de l'urémie et de l'hémoglobine. Une comparaison de ces deux scores a été faite pour déterminer le pronostic et l’évolution de cette pathologie. 46,3% des dossiers n'avaient pas de valeur d'urémie ni de l'hémoglobine. Les 185 patients retenus, avaient une moyenne d’âge de 48,2 ± 18,2 ans et une prédominance masculine (72,4%). Le risque clinique était élevé dans 62,7% des cas, dont 59,4% ont bénéficié d'une transfusion. Une mortalité de 9,7% a été observée. Le score de Blatchford était plus prédictif du risque clinique par rapport au score de Rockall initial (AUROC = 0,82 vs 0,64; p < 0,0001), mais aussi de la nécessité d'une transfusion (AUROC = 0,79 vs 0,62; p= 0,00013). L'instauration d'un score clinico-biologique est assez limitée par faute de moyens, alors que le score de Blatchford aiderait en matière de pronostic des hémorragies digestives hautes.

## Introduction

Les hémorragies digestives hautes sont un motif très fréquent d'hospitalisation, responsable d'une morbidité et d'une mortalité élevées [1–3]. Actuellement, il est recommandé d'utiliser des scores pronostiques pour assister les praticiens afin de classifier les patients à faible risque et élevé et éviter ainsi l'admission en unité de soins intensifs d'emblée voire l'hospitalisation systématique [2, 4–6]. Parmi ces scores: le score de Rockall qui peut être subdivisé en score de Rockall initial (IRS) avec des items uniquement cliniques, lequel est dit complet, lorsque s'ajoutent les données endoscopiques (Tableau 1) [7]. Egalement, le score de Glasgow-Blatchford (GBS) qui est un score clinico-biologique, dont l'avantage est qu'il peut être réalisé dès l'admission du patient, bien avant l'endoscopie digestive (Tableau 2) [8]. L'objectif de cette étude a été d’évaluer l'utilisation de deux scores pronostiques, à savoir: le score de Rockall initial et celui de Glasgow-Blatchford et de les comparer dans l’évaluation du pronostic et de l’évolution des hémorragies digestives hautes (HDH) non variqueuses.

**Tableau 1:** Score de Rockall

	0	1	2	3		
Age	< 60 ans	60- 79 ans	>80 ans		**Score initials**	**Score de Rockall complet**
Signe de choc	Pas de signe choc	« Tachycardie » Pouls ≥100 bpm PAS ≥ 100mmHg	« Hypotension » Pouls ≥100 bpm PAS ≤ 100mmHg			
Comorbidité	Non	Non	Cardiopathie ischémique, insuffisance cardiaque. Toute comorbidité majeure	Insuffisance rénale, hépatique. Cancer généralisé		
**Diagnostic endoscopique**	Mallory Weiss. Absence de lésion et de stigmates de saignement récent	Tout autre diagnostic	Lésions malignes		Critères ajoutés pour le score complet	
**Signe en faveur d'un saignement récent**	*Forrest III/ IIc:* Absence d'hémorragie, lésions noirâtres de la base de l'ulcère		*Forrest Ia, IIa ou IIb:* Caillot adhérent visible, vaisseau en cours de saignement			

Rockall TA, Devlin HB, Logan RFA, Northfield TC, for the National Audit of Acute Upper Gastrointestinal Haemorrhage. Selection of patients for early discharge or outpatient care after acute upper gastrointestinal haemorrhage. Lancet. 1996; 347 (9009): 1138-1140

**Tableau 2:** Score de Glasgow-Blatchford

Items	Valeurs	Points
Urée (mmol/L)	6,5 à 8,0	2
	8,0 à 10,0	3
	10,0 à 25,0	4
	≥ 25,0	6
Hémoglobine (g/dL) *Homme*	12,0 à 13,0	1
	10,0 à 12,0	3
	< 10,0	6
Hémoglobine (g/dL) *Femme*	10,0 à 12,0	1
	< 10,0	6
Pression artérielle systolique (mmHg)	100 – 109	1
	90 - 99	2
	< 90	3
**Autres marqueurs**		
Pouls ≥ 100 bpm		1
Melaena		1
Syncope		2
Hépatopathie		2
Insuffisance cardiaque		2

Blatchford Oliver, Murray William R, Blatchford Mary. A risk score to predict need for treatment for uppergastrointestinal haemorrhage. Lancet. 2000; 356(9238):1318-1321.

## Méthode

Nous avons réalisé une étude observationnelle, prospective, sur une période de douze mois, entre janvier et décembre 2011, au Service de Réanimation Chirurgicale du Centre Hospitalo-Universitaire d'Antananarivo - Madagascar. Les patients âgés de plus de 18 ans, ayant présenté une hémorragie digestive haute ont été étudiés. La suspicion, l’étiologie variqueuse et l'association de varices après anamnèse et exploration, de même que les patients ayant présenté une hémorragie digestive lors de leur hospitalisation ont été exclus. Devant l'urgence de la pathologie, les premiers soins étaient administrés en même temps que la prise des paramètres cliniques et les prélèvements sanguins en vue d'examen biologique. Les données cliniques et biologiques, plus particulièrement les valeurs de l'urémie et du taux d'hémoglobine ont été recueillies, afin de calculer les différents scores. Ceux dont les données manquaient étaient également exclus de l’étude. Une comparaison des scores de Rockall initial et de Glasgow Blatchford pour chaque patient, a été faite afin de déterminer lequel était plus à même de déterminer le pronostic de ces patients, notamment en termes de transfusion sanguine, persistance ou récidive de l'hémorragie, nécessité d'un traitement chirurgical ou décès du patient; lesquels déterminaient le risque clinique. La récidive de l'hémorragie était définie par une réadmission dans le service dans les 30 jours après le transfert en secteur, une hémorragie après lavement propre et/ou selle normale; la persistance de l'hémorragie par l'activité continue de l'hémorragie sous transfusion avec pour objectif une hématocrite à 30% ou une hémorragie associée à une chute de deux points du taux d'hémoglobine en 24 heures. Des tests de régression logistique multiple, de régression linéaire, l'analyse des courbes ROC, le test du Chi2 pour la comparaison des aires sous les ROC (AUROC) ont été effectués (SigmaPlot 10.0^®^).

## Résultats

**Figure 1:**
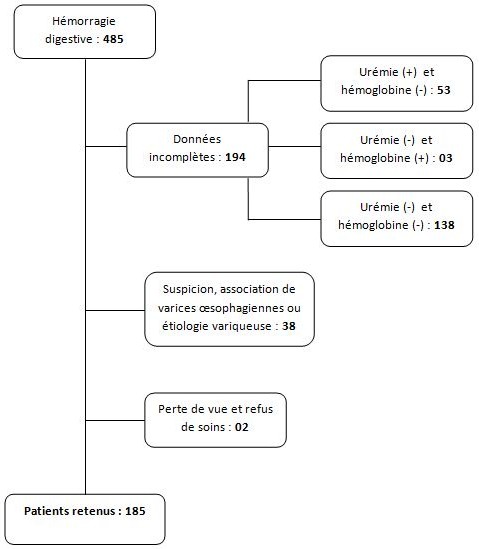
Sélection des patients lors de notre étude

**Figure 2:**
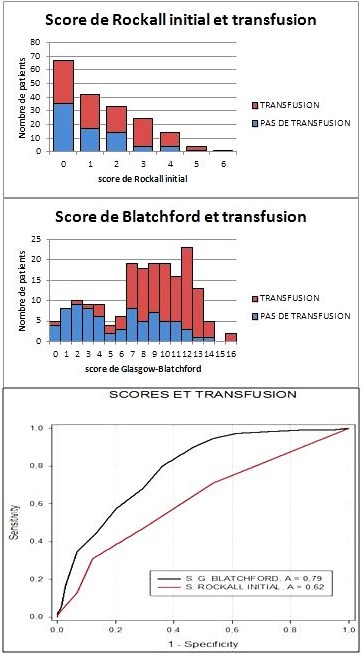
Transfusion et scores (comparaison des proportions de transfusion selon les scores, ainsi que les courbes ROC relatives à ces deux scores et à la transfusion)

**Figure 3:**
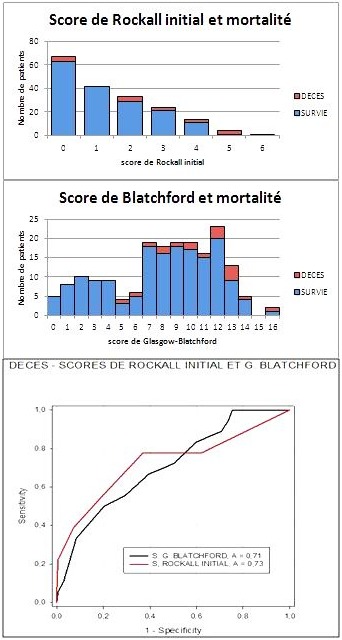
Décès et scores (comparaison de la survenue du décès selon les scores, ainsi que les courbes ROC relatives à ces deux scores et au décès)

Sur 419 patients admis pour hémorragie digestive haute, seuls 185 patients ont été inclus après exclusion de 234 dossiers (Figure 1). L’âge moyen de la population était de 48,0±18,2 ans, avec une prédominance masculine (sex ratio 2,63). Les patients étaient de la classe ASA I (sans comorbidité notable) dans 70,8% des cas, 25,9% ont présenté une atteinte modérée d'une grande fonction (ASA II) et 3,2% une atteinte sévère d'une grande fonction, n'entraînant pas d'incapacité (ASA III). La présentation de l'hémorragie était dans 43,2% une hématémèse associée à un méléna, une hématémèse seule dans 24,9% et d'un méléna seul dans 31,9%. L'accident était inaugural dans 71,3%. La durée de séjour moyen était de 5,0±3,9 jours. Le risque clinique élevé; autrement dit, la réalisation d'une transfusion sanguine, le recours au traitement chirurgical, la persistance ou la récidive de l'hémorragie ou le décès; était présent dans 62,7% des cas. Le score de Rockall initial variait de 0 (36,2%) à 6 (0,5%) avec une médiane de 1. Le score de Glasgow-Blatchford variait de 0 (2,7%) à 16 (1,1%) avec une médiane de 9. Cent-dix patients (59,4%) ont été transfusés (Figure 2). Un taux de mortalité de 9,7% a été retrouvé, dont 22,2% d'une décompensation de leur pathologie sous-jacente (Figure 3).

Après régression logistique, le score de Glasgow-Blatchford était plus prédictif, de manière significative, de la nécessité d'une transfusion sanguine (OR = 1,392 - IC95%: (<1,245-1,555), p < 0,001 contre OR = 1,044 - IC95%: (0,802-1,360), p = 0,747, pour le score de Rockall initial). Aucun des deux scores ne pouvait prévoir ni une éventuelle intervention chirurgicale, ni la récidive ni la persistance du saignement (p > 0,05). Par contre, le score de Rockall déterminait mieux la mortalité (OR = 1,736 - IC95%: (1,202-2,509), p = 0,003 contre OR = 1,152 - IC95%: (0,965-1,202), p = 0,118, pour le GBS). Après analyse des courbes ROC, le score de Blatchford était plus spécifiquement prédictif du risque clinique, par rapport au score de Rockall initial (AUROC GBS: 0,82 - AUROC IRS: 0,64; p < 0,0001). Du point de vue transfusion sanguine, les AUROC des scores de Blatchford et de Rockall initial étaient de 0,79 et 0,62 respectivement (p= 0,00013) avec une spécificité plus importante pour le score de Blatchford (Figure 2, Tableau 3). Par contre, aucune différence significative n'a été constatée entre le score de Rockall initial et le score de Blatchford en termes de mortalité, AUROC étant de 0,71 et de 0,73 respectivement (p = 0,81) (Figure 3). Aucune relation significative n'a été retrouvée entre les deux scores et la durée de séjour en réanimation (p = 0,146 pour le GBS et 0,976 pour le score de Rockall initial).

**Tableau 3:** Sensibilité et spécificité des scores de Glasgow-Blatchford et de Rockall initial

		Score de Glasgow-Blatchford		Score de Rockall initial	
		Sensibilité (%)	Spécificité (%)	Sensibilité (%)	Spécificité (%)
**Risque clinique**	> 0	99,1	5,8	70,7	47,8
	> 1	99,1	17,4	49,1	72,5
	> 2	98,3	30,4	31,0	89,9
	> 3	97,4	42,0	12,9	94,2
	> 4	94,8	50,7	4,3	100,0
	> 5	93,1	53,6	0,9	100,0
	> 6	90,5	58,0	-	-
	> 7	81,0	69,6	-	-
	> 8	68,1	73,9	-	-
	> 9	56,9	82,6	-	-
	> 10	44,0	88,4	-	-
	> 11	33,6	94,2	-	-
	> 12	16,4	98,5	-	-
	> 13	6,0	100,0	-	-
	> 15	1,7	100,0	-	-
**Transfusion**	> 0	99,1	5,3	70,9	46,7
	> 1	99,1	16,0	48,2	69,3
	> 2	98,2	28,0	30,9	88,0
	> 3	97,3	38,7	12,7	93,3
	> 4	94,5	46,7	3,6	98,7
	> 5	92,7	49,3	0,9	100,0
	> 6	90,0	53,3	-	-
	> 7	80,0	64,0	-	-
	> 8	68,2	70,7	-	-
	> 9	57,3	80,0	-	-
	> 10	44,5	86,7	-	-
	> 11	34,5	93,3	-	-
	> 12	16,4	97,3	-	-
	> 13	5,5	98,7	-	-
	> 15	1,8	100,0	-	-

## Discussion

Au décours de cette étude, nous avons pu constater que le recours aux scores de Rockall initial et de Glasgow-Blatchford semble intéressant afin de déterminer le profil pronostique et évolutif des hémorragies digestives hautes non variqueuses. Néanmoins, l’établissement de ce score reste encore assez difficile, dans notre milieu, du fait de la non accessibilité des examens biologiques, par certains patients faute de moyens financiers (46,3% d'exclusion par défaut de valeur de l'urémie et/ou du taux de l'hémoglobine); car seul le patient pourvoit à ses dépenses hospitalières. Pourtant la valeur de l'urémie est un marqueur important dans les hémorragies digestives hautes, qui reflète la digestion de l'hémoglobine [8]. Par la détermination de ces scores, il serait ainsi plus facile de mieux orienter les patients dont l'admission dans notre milieu se fait systématiquement en réanimation chirurgicale.

Le score de Rockall, a été établi pour mieux déterminer le risque de resaignement et de mortalité, notamment lorsque celui-ci est complet [5, 7, 9]. Dans son article princeps, Blatchford a établi un score qui déterminait mieux plus le risque clinique et qui était plus corrélé avec la sévérité de l'hémorragie, par rapport au score de Rockall, de par la détermination de la durée de séjour ainsi que de la nécessité d'une thérapeutique interventionnelle: transfusionnelle ou endoscopique [5, 8, 10]. En termes de scores pronostiques, le score de Blatchford tend à être de plus en plus utilisé en comparaison avec le score de Rockall [4, 10, 11].

Le score de Blatchford s'avère plus prédictif en termes de pronostic, notamment dans la nécessité d'une transfusion. Il en est de même dans la prédiction d'une intervention chirurgicale ou endoscopique, par rapport au score de Rockall initial, mais identique au score de Rockall complet [2, 4, 10, 12]. Des résultats similaires ont été retrouvés dans notre étude, mise à part l'intervention endoscopique. Cette supériorité du GBS, dans certaines études, dans la détermination du risque clinique ou la nécessité de transfusion a été également retrouvée en comparaison avec le score de Rockall complet, rendant celui-ci intéressant, notamment dans le cadre d'une politique d’économie de produits sanguins labiles, intéressantes dans un pays en développement comme le nôtre [3, 4, 10, 11].

En termes de récidive, Blatchford révèle son score plus prédictif [8]. Les récentes études n'abondent pas en ce sens. En effet, ce score serait moins prédictif que le score de Rockall complet qui détermine mieux ce risque [11, 13, 14]. Même que parfois, aucun des deux scores ne puissent prévoir le risque de resaignement, tel que dans une étude danoise comparant les scores de Rockall, de Blatchford et d'autres scores qui trouve qu'aucun d'entre eux n’étaient contributifs, voire même que le score de Rockall initial avait une très faible valeur prédictive [6] tout comme nous avions pu retrouver dans nos résultats.

Le score ayant le plus évalué significativement le risque de mortalité était le score de Rockall [3, 7]. Au fil des études, la spécificité de ce score a tendance à diminuer. Soit, les scores de Rockall et de Blatchford ont les mêmes valeurs prédictives [4, 12]; soit le score de Blatchford est supérieur à celui de Rockall dans la prédiction de la mortalité [3]. Comparé à plusieurs autres scores, entre autre le score de Rockall complet, le GBS est peu prédictif de la mortalité [6]. Le score de Rockall complet était plus prédictif par rapport au score de Blatchford et quasiment similaire au score de Rockall initial [11]. Parfois, selon les travaux, il se trouve qu'aucun de ces deux scores ne soient spécifiques de la mortalité [13, 14].

Les scores pronostiques permettraient également de déterminer la nécessité d'une hospitalisation ou d'un retour à domicile, surtout dans les pays nantis tels que les Etats-Unis [2, 4]. Le GBS établi avait une corrélation significative avec la durée de séjour et permettait une meilleure orientation des patients, voire une prise en charge en ambulatoire [8, 13]. Dans notre étude, aucune corrélation significative n'a été retrouvée entre les deux scores ainsi que la durée de séjour. Ainsi, on pourrait dire que se baser sur ces scores permettrait une meilleure orientation du patient, qui ne se ferait pas systématiquement en réanimation chirurgicale - mais d'autres études seraient nécessaires pour analyser cette hypothèse. Malgré tout, la décision des praticiens concernant l'hospitalisation ou non du patient est toujours importante [1, 2].

Le caractère monocentrique de notre étude, le faible échantillon dû à un taux élevé d'exclusion par manque de données en particulier les valeurs d l'urémie et parfois de l'hémoglobine limitent fortement notre étude; mais permettent d’établir qu'il est encore assez difficile d'obtenir toutes ces informations dû au faible revenu de la plupart des patients dans un pays en développement. De plus, une analyse comparative avec le score de Rockall complet aurait été plus intéressante, pour déterminer la supériorité du score de Blatchford, tout comme l'ont retrouvé d'autres études [4, 11]. Comparer les deux scores afin de prédire le risque interventionnel endoscopique aurait aussi été nécessaire, cependant, l'accès à cette thérapeutique s'avère encore inexistante dans notre milieu. Cette étude serait alors contributive dans le sens où l'utilisation de ces scores est toutefois réalisable dans notre pratique, notamment le score de Blatchford qui pourrait mieux déterminer les patients à faible et haut risque et ainsi, en améliorer al prise en charge voire réduire les dépenses hospitalières.

## Conclusion

L'utilisation de scores pronostiques devant une hémorragie digestive haute non variqueuse trouve son intérêt notamment dans l’évaluation du risque clinique et de la mortalité. Leur recours devrait être plus systématique, le score de Rockall initial étant un score basé sur des éléments essentiellement cliniques et celui de Glasgow Blatchford, basé sur des éléments clinico-biologiques. Aussi pour mieux cadrer le patient présentant une hémorragie digestive, il serait essentiel d'effectuer un examen clinique minutieux et de réaliser au minimum une numération formule sanguine (avec taux d'hémoglobine) et la mesure de l'urémie.

## References

[1] Farooq Farees T, Lee Michael H, Das Ananya, Dixit Rahul, Wong Richard CK (2012). Clinical triage decision vs risk scores in predicting the need for endotherapy in upper gastrointestinalbleeding. Am J Emerg Med.

[2] Chandra Subhash, Hess Erik P, Agarwal Dipti, Nestler David M, Montori Victor M, Wong Kee Song Louis M, Wells George A, Stiell Ian A (2012). External validation of the Glasgow-Blatchford Bleeding Score and the Rockall Score in the US setting. Am J Emerg Med.

[3] Chiu Phillip WY, Sung Joseph JY (2010). Acute nonvariceal upper gastrointestinal bleeding. Cur Opin Gastroenterol..

[4] Stanley Adrian J, Dalton Harry R, Blatchford Oliver, Ashley Dawn, Mowat Craig, Cahill Aidan, Gaya Daniel R, Thompson Emma, Warshow Usama, Hare Nikki, Groome Max, Benson George, Murray William (2011). Multicentre comparison of the Glasgow Blatchford and Rockall scores in the prediction of clinical end-points after upper gastrointestinal haemorrhage. Aliment Pharmacol Ther.

[5] Badel Stéphane, Dorta Gian, Carron Pierre-Nicolas (2011). Hémorragies digestives hautes: utilité des scores pronostiques. Rev Med Suisse.

[6] Laursen Stig Bobjerg, Hansen Jane Møller, De Muckadell Ove B Schaffalitzky (2012). The Glasgow Blatchford score is the most accurate assessment of patients with upper gastrointestinal hemorrhage. Clin Gastroenterol Hepatol.

[7] Rockall TA, Devlin HB, Logan RFA, Northfield TC, for the National Audit of Acute Upper Gastrointestinal Haemorrhage (1996). Selection of patients for early discharge or outpatient care after acute upper gastrointestinal haemorrhage. Lancet..

[8] Blatchford Oliver, Murray William R, Blatchford Mary (2000). A risk score to predict need for treatment for uppergastrointestinal haemorrhage. Lancet.

[9] Chen I-Chuan, Hung Ming-Szu, Chiu Te Fa, Chen Jih-Chang, Hsiao Chen Ting (2007). Risk scoring systems to predict need for clinical intervention for patients with nonvariceal upper gastrointestinal tract bleeding. Am J Emerg Med.

[10] Farrell Richard J, Alsahli Mazen, LaMont J Thomas (2000). Is successful triage of patients with upper-gastrointestinal bleeding possible without endoscopy’. Lancet.

[11] Dicu Daniela, Pop Felicia, Ionescu Daniela, Dicu Tiberius (2013). Comparison of risk scoring systems in predicting clinical outcome at upper gastrointestinal bleeding patients in an emergency unit. Am J Emerg Med.

[12] Bjorkman David J (2011). Which scoring system best predicts outcomes of upper gastrointestinal bleeding’ JW Gastroenterol. http://www.jwatch.org/jg201107290000001/2011/07/29/which-scoring-system-best-predicts-outcomes.

[13] Lahiff Connor, Shields William, Cretu Ion, Mahmud Nasir, McKiernan Susan, Norris Suzanne, Silke Bernard, Reynolds John V, O'Toole Dermot (2012). Upper gastrointestinal bleeding: predictors of risk in a mixed patient group including variceal and nonvariceal haemorrhage. Eur J Gastroenterol Hepatol.

[14] Kim Beom Jin, Park Moon Kyung, Kim Sang-Jung, Kim Eun Ran, Min Byung-Hoon, Son Hee Jung, Rhee Poong-Lyul, Kim Jae J, Rhee Jong Chul, Lee Jung Haeng (2009). Comparison of Scoring Systems for the Prediction of Outcomes in Patients with Nonvariceal Upper Gastrointestinal Bleeding: A Prospective Study. Dig Dis Sci.

